# Global Co-Occurrence Feature and Local Spatial Feature Learning for Skeleton-Based Action Recognition

**DOI:** 10.3390/e22101135

**Published:** 2020-10-06

**Authors:** Jun Xie, Wentian Xin, Ruyi Liu, Qiguang Miao, Lijie Sheng, Liang Zhang, Xuesong Gao

**Affiliations:** 1 School of Computer Science and Technology, Xidian University, Xi’an 710071, China; wtxin@stu.xidian.edu.cn (W.X.); qgmiao@xidian.edu.cn (Q.M.); ljsheng@xidian.edu.cn (L.S.); liangzhang@xidian.edu.cn (L.Z.); 2 State Key Laboratory of Digital Multimedia Technology, Hisense Co., Ltd., Qingdao 266071, China; gaoxuesong@hisense.com

**Keywords:** skeleton-based action recognition, graph convolutional network, feature fusion

## Abstract

Recent progress on skeleton-based action recognition has been substantial, benefiting mostly from the explosive development of Graph Convolutional Networks (GCN). However, prevailing GCN-based methods may not effectively capture the global co-occurrence features among joints and the local spatial structure features composed of adjacent bones. They also ignore the effect of channels unrelated to action recognition on model performance. Accordingly, to address these issues, we propose a Global Co-occurrence feature and Local Spatial feature learning model (GCLS) consisting of two branches. The first branch, based on the Vertex Attention Mechanism branch (VAM-branch), captures the global co-occurrence feature of actions effectively; the second, based on the Cross-kernel Feature Fusion branch (CFF-branch), extracts local spatial structure features composed of adjacent bones and restrains the channels unrelated to action recognition. Extensive experiments on two large-scale datasets, NTU-RGB+D and Kinetics, demonstrate that GCLS achieves the best performance when compared to the mainstream approaches.

## 1. Introduction

In the field of computer vision, human action recognition plays an important role, with the purpose of predicting the action classes of videos. This is a fundamental yet challenging task that provides technical support for downstream applications such as video surveillance, human-machine interaction, video retrieval, and game-control [1,2,3]. Due to their effectiveness in action representation, their robustness against sensor noise, and their efficiency in computation and storage, action recognition methods based on skeleton data have been widely investigated and have attracted considerable attention.

However, there are three shortcomings in existing works. (1) Because ST-GCN [4] may not adequately capture the dependency between far-apart joints [5], it is unable to effectively extract the global co-occurrence features of actions. (2) Since 1×1 convolution cannot consider the relationship between each vertex and its surrounding vertices, these related works [4,6,7] may not effectively obtain the spatial features composed of adjacent vertices. (3) These works [4,6,7] expand the number of channels per-vertex as the number of network layers increases. Thus, there may be some channels that are unrelated to the action recognition in the hundreds of channels after expansion, which may also affect the model performance.

**Figure 1:**
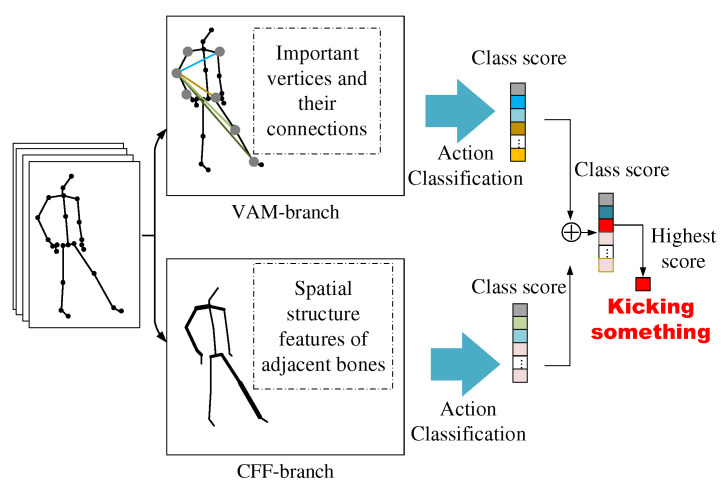
The overall structure of GCLS. The VAM-branch captures the global co-occurrence features composed of important vertices and the connections among them, while the CFF-branch captures the spatial structural features formed by adjacent bones. The two branches are integrated by the voting mechanism, and the action category with the highest number of votes is taken as the final action category of the current action. In the VAM-branch, an important vertex is represented as a dark gray circle; in the CFF-branch, the thickness of the bone is determined by its feature map.

**Figure 2:**
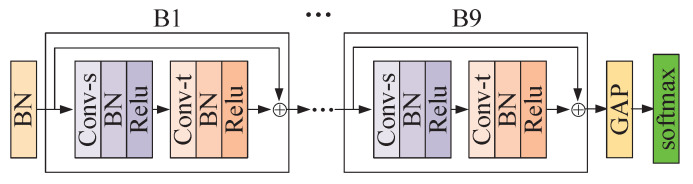
Illustration of the network framework of the branch of GCLS. Conv-s and Conv-t denote the spatial GCN and temporal GCN respectively. B1,⋯,B9 denote the nine basic blocks and GAP denotes a global average pooling layer. For VAM-branch, Conv-s is implemented by VAM. For CFF-branch, Conv-s is implemented by CFF.

**Figure 3:**
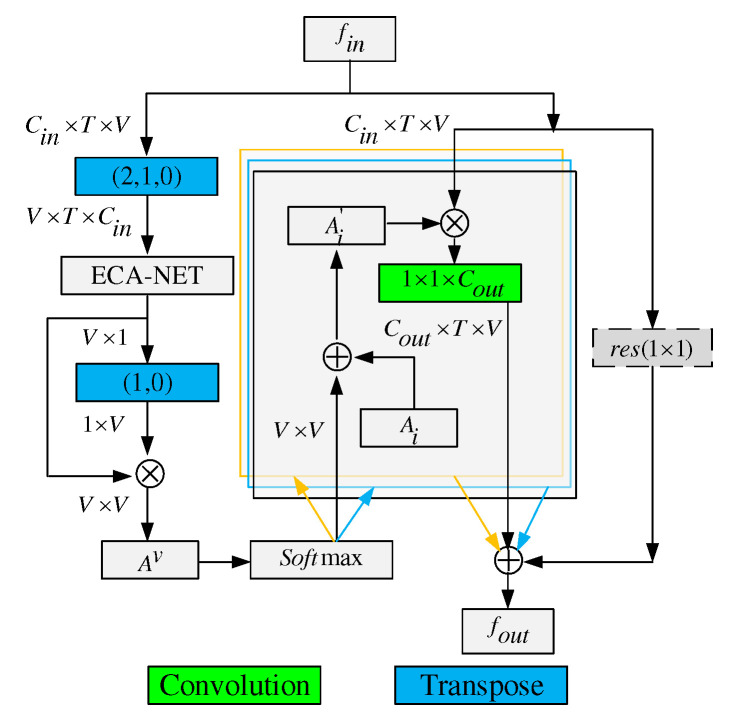
Illustration of spatial convolution for VAM-branch. The adjacency matrix for the skeleton is divided into three submatrices, i.e.,$A_{i}$(i=1,2,3). Green blocks represent convolution layers, where the last dimension denotes the number of output channels. A transpose layer permutes the dimensions of the input tensor according to the order parameter. ⊗ denotes the element-wise summation. ⊗ denotes the matrix multiplication. The residual box (dotted line) is only needed when $C_{in}$ is not the same as $C_{out}$.

**Figure 4:**
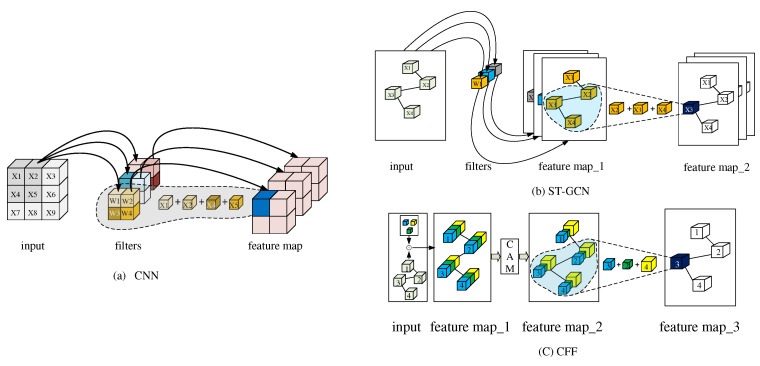
Feature fusion of different models. (**a**) Feature fusion of CNN. (**b**) Feature fusion of ST-GCN. (**c**) Feature fusion of CFF. Blue cube, green cube, and yellow cube denote filter1 (w1), filter2 (w2), and filter3 (w3), respectively. ⊙ denotes convolution computation.

**Figure 5:**
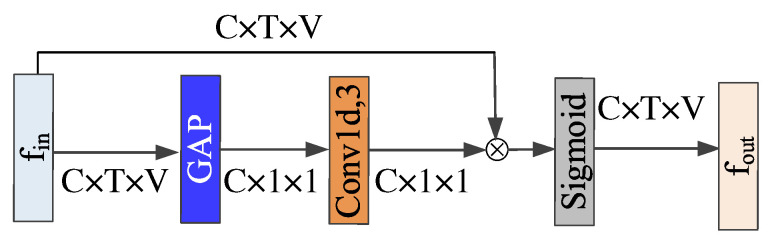
Illustration of CAM. Blue blocks are global average pooling layers. Light brown blocks are 1d convolution layers, where the last dimension denotes the size of the convolution kernel. ⨂ denotes element-wise product.

**Figure 6:**
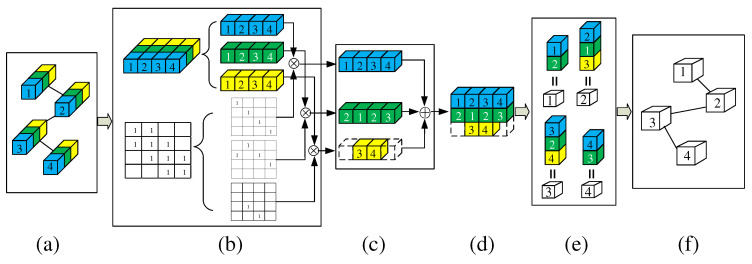
Illustration of CFA. This figure focuses on the Cross-kernel feature Fusion Algorithm (CFA), so the CAM module is omitted. Plot (**a**) is equivalent to feature map_1 in Figure 4c. In plot (**b**), the graph in the plot (**a**) is represented by its adjacency matrix and the features of all nodes. ⨂ denotes matrix multiplication. Plot (**c**) shows the results of multiplying three groups of features and three matrices. Plot (**d**) means that the three results are added together. Plot (**e**) shows the composition of the features of each root node, where the four numbered cubes without color represent four root nodes. Plot (**f**) represents the update graph composed of four root nodes in Plot (**e**).

To solve the above problems, we propose a Global Co-occurrence feature and Local Spatial feature learning model (GCLS), which consists of two branches. The Vertex Attention Mechanism branch (VAM-branch) can extract the global co-occurrence features of actions effectively, while the Cross-kernel Feature Fusion branch (CFF-branch) extracts local spatial structure features composed of adjacent bones and restrains the channels that are unrelated to action recognition. The two branches are integrated by the voting mechanism. Figure 1 describes the overall structure of GCLS, where the dark gray circle denotes the important joints and the thickness of the bone is determined by its feature maps (feature map is the result of a convolution of input data by the neural network) for action recognition. Both branches are based on the same network framework that is shown in Figure 2. The network framework is composed of nine basic modules, each of which is composed of temporal convolution and spatial convolution. The difference between the two branches is that the spatial convolution module uses VAM and CFF respectively. For VAM-branch, our idea to obtain the global co-occurrence features of action through an attention mechanism. The co-occurrence feature is combined with the adjacency matrix of the skeleton graph to form a new adjacency matrix, which is utilized to capture the dependency between far-apart joints. Figure 3 shows this process. For CFF-branch, first, we analyze the differences of feature fusion process between prevailing GCN-based methods and CNN, so as to obtain the limitations of previous related work. Based on these limitations, the CFF is proposed. This process of comparison and analysis is shown in Figure 4. The CFF is made up of Channel Attention Module (CAM) and Cross-kernel feature Fusion Algorithm (CFA). We first introduce a CAM to suppress channels that are not associated with action recognition, which is shown in Figure 5. Then, we propose a CFA to overcome the limitations of previous related work, which improves the ability to capture the spatial relationship of adjacent bones. Figure 6 shows the detailed implementation of the CFA. Inspired by the feature learning framework [8], we train the two branches of our network (VAM-branch and CFF-branch) with joints and bones as input data respectively. The difference is that the feature learning framework [8] is based on CNN, while our model is based on GCN. In the verification stage, the two branches vote on the respective prediction action classes, and the action class with the highest number of votes was taken as the final action class.

To verify the superiority of the proposed model, extensive experiments are conducted on two large-scale datasets: NTU-RGB+D and Kinetics. Our model achieves state-of-the-art performance on both of these datasets. The specific contributions of this paper can be summarized as follows:

• We construct a new adjacency matrix through Vertex Attention Mechanism (VAM) to extract the global co-occurrence features of actions. To the best of our knowledge, this is the first research attempt to exploit the VAM of GCN for the global co-occurrence features of actions.
• We propose a Cross-kernel feature Fusion (CFF), instead of using the traditional feature fusion based on the same convolution kernel. This novel feature fusion method significantly improves the ability to capture spatial features of adjacent bones.
• On two large-scale datasets, NTU-RGB+D and Kinetics, the experimental results demonstrate that GCLS achieves superior performance compared to existing state-of-the-art methods.

The remainder of this paper is organized as follows. In Section 2, related work is discussed. Section 3 covers the necessary background material for the rest of the paper. In Section 4, we explain the proposed methodology, and we describe and discuss extensive experimental results in Section 5. Finally, the conclusions are presented in Section 6.

## 2. Related Work

This section reviews related work on: skeleton-based action recognition and the attentional mechanism in graph convolution network.

### 2.1. Skeleton-Based Action Recognition

According to the different models used, skeleton-based action recognition methods can be divided into two categories: namely, traditional methods and methods based on deep learning. The traditional methods realize action recognition by capturing the intuitive patterns of physical action, such as joint velocity and skeletal rotation angle [9,10,11]. Moreover, deep learning-based methods can be further divided into RNN-based, CNN-based, and GCN-based. RNN-based methods model the skeleton data using a sequence of vectors, which are then fed into the RNN model to realize action recognition [12,13,14,15,16,17,18,19,20,21]. CNN-based methods convert skeleton data into images and then feed the images into the CNN model to realize action recognition [22,23,24,25,26,27,28,29,30]. The traditional methods need to design features by hand, which has become an important bottleneck in their development. As the skeleton is essentially the graph of non-Eulerian space, CNNs and RNNs are unable to represent the structural features of the skeleton’s joints very well. Recently, Yan et al. [4] directly modeled the skeleton data as a graph structure using a Spatial-Temporal Graph Convolutional Networks (ST-GCN), which solves the existing problems in traditional methods, CNN-based and RNN-based, and achieves good action recognition results. Inspired by ST-GCN, the latest research works [6,7] further proposed the parameterization of the skeleton topology, which makes the topology structure learn together with other model parameters. Therefore, the topological structure of the skeleton varies with the sample and network layers, which further increases the accuracy of action recognition. In this work, we adopt the graph-based approach for action recognition. Different from any prevailing GCN-based methods, we learn the global co-occurrence features and local spatial structure features from data, which captures useful full information about actions.

### 2.2. Attentional Mechanism in Graph Convolution Network

To further improve the performance of GCN, attention mechanisms are introduced to GCN [31,32]. The attention mechanism can make the algorithm or model focus on relatively critical from all inputs. Inspired by this, Velickovic et al. [32] improved the performance of the graph node classification model through attention mechanism and achieved state-of-the-art performance. Sankar et al. [33] introduce a self-attention mechanism in the study of dynamic graph representation learning and get superior results on link prediction tasks. Nonetheless, our work is different since we construct a new adjacency matrix through attention mechanism, while others are to compute the importance weights either for frames or different feature representations.

## 3. Background

In this section, we cover the background material necessary for the rest of the paper.

### 3.1. Efficient Channel Attention (ECA)

ECA-Net [34] consists of two steps: namely, squeeze and appropriate cross-channel interaction. In the squeeze stage, the dimension number of features is compressed to the dimension represented by the channel through a global pooling operation. Let the number of channels of a feature be *C*, while the number of elements in each channel is M×N; that is, the number of elements of the feature is C×M×N. After the squeeze, the number of elements of this feature is *C*. The squeezing process can be written as:

(1) $$\begin{array}{c} h^{c}=\frac{1}{M\times N}\sum_{i=1}^{M}\sum_{j=1}^{N}c_{x}\left(i,j\right) \end{array}$$

where $c_{x}$ denotes the $c^{\mathrm{th}}$ channel in *x*. This equation describes the squeeze process of the $c^{\mathrm{th}}$ channel in *x*. After squeeze, this feature is: $\left\{h^{1},h^{2},\cdots ,h^{C}\right\}$.

In the appropriate cross-channel interaction stage, a local cross-channel interaction strategy without dimension reduction is used to learn the weight of each channel. More details may be referred to [34]. As far as we know, it is the first time that to apply ECA-Net to the research field of action recognition.

### 3.2. Graph Convolutional Network

In this section, we first introduce the definition of graph and skeleton data, and then give a brief introduction to graph convolution network (GCN).

#### 3.2.1. Graph and Skeleton Data

**Graph:** By definition, a weighted directed graph can be represented by G=(V,X,A) where $V=\{v_{1},v_{2},\cdots v_{V}\}$ is the set of nodes and $A\in {\left\{0,1\right\}}^{V\times V}$ is the adjacency matrix. If there is an edge from $v_{i}$ to $v_{j}$, then $A_{ij}=1$ otherwise $A_{ij}=0$. $X\in R^{V\times C}$ represents the features of each node, *C* is the number of feature channels and *V* is the number of nodes.

**Skeleton Data:** Since the skeleton data comes from a video clip, the skeleton data is composed of several frames, and the skeleton in each frame constitutes a graph. This graph uses joints as vertices and bones as edges. If the number of frames is *T*, then according to the definition of graph, $X\in R^{T\times V\times C}$ represents the features of each node. In other words, skeleton data can be described as a tensor with the shape T×V×C.

#### 3.2.2. Graph Convolutional Network (GCN)

Here, we provide a brief introduction to GCN [35]. GCN, a widely used Graph Neural Networks (GNN) architecture, is chosen as one of the key building blocks in our work. At each layer of the GCN, the convolution operation is applied to each node as well as its neighbors and the new representation of the $i^{\mathrm{th}}$ node is computed through the function:

(2) $$\begin{array}{c} x_{i}^{l+1}=\sigma \left(\sum_{j\in D_{i}}\frac{1}{C_{i}}x_{j}^{l}w_{ij}^{l}\right) \end{array}$$

where $x_{j}^{l}\in R^{d}$ represents the $j^{\mathrm{th}}$ node features at the layer *l*, σ is a nonlinear activation function, *d* represents the number of feature channels of the node, $C_{i}$ is the cardinality of the $D_{i}$ that represents a set consisting of node *i* and its neighbors, $w_{ij}^{l}$ is a trainable weight between the $j^{\mathrm{th}}$ node and the $i^{\mathrm{th}}$ node at the layer *l*. In Equation (2), $D_{i}$ is also called the *neighbor set* and $x_{i}$ is called the *root node*. The new representations of all node are computed through the function:

(3) $$\begin{array}{c} X^{l+1}=\sigma \left(\Lambda^{-\frac{1}{2}}A\Lambda^{-\frac{1}{2}}X^{l}W^{l}\right) \end{array}$$

where $\Lambda^{ii}=\sum_{j}A^{ij}$, *A* represents the adjacency matrix of a graph. In ST-GCN [4], each node and its neighbors are divided into three categories according to the vertex partition strategy, so the adjacency matrix of the graph is also divided into three parts: $A_{1}$, $A_{2}$ and $A_{3}$. So Equation (3) can be rewritten as:

(4) $$\begin{array}{c} X^{l+1}=\sigma \left(\sum_{j=1}^{3}\Lambda_{j}^{-\frac{1}{2}}A_{j}\Lambda_{j}^{-\frac{1}{2}}X^{l}W^{l}\right) \end{array}$$

## 4. Methodology

As shown in Figure 1, GCLS consists of a VAM-branch and a CFF-branch. In this section, we first describe the network structure of branches and basic modules, then describe the implementation details of the spatial GCN of different branches; Finally, we describe how the two branches are integrated by the voting mechanism.

### 4.1. Network Structure of Branches and Basic Modules

Figure 2 shows a network framework, containing nine basic modules whose number of output channels are 64, 64, 64, 128, 128, 128, 256, 256, and 256, respectively. The basic module consists of a temporal GCN and a spatial GCN, both of which are followed by a BN layer and a ReLU layer. We add a batch normalization (BN) layer for normalization at the beginning and a softmax layer for prediction at the end. A global average pooling layer is inserted between the 9th basic module and the softmax layer to map the feature maps of different sizes to the feature maps of the same sizes. To stabilize the training, a residual connection is added for each basic module. We further build the two branches of GCLS with this framework. The difference between the two branches is that one branch uses VAM for spatial GCN while the other uses CFF. In this paper, the temporal GCN of the basic module is the same as the temporal GCN of ST-GCN. Next, we will introduce VAM and CFF in more detail.

### 4.2. Vertex Attention Mechanism (VAM)

Previous related works may not effectively identify many human movements that need to be completed by the cooperative movement of far-apart joints. This is caused by the fact that when GCN tries to aggregate the wider-range features with hierarchical GCNs, the joint features may be weakened during long diffusion [5]. This phenomenon indicates that these human movements cannot be effectively identified. Namely, GCN is unable to effectively extract the global co-occurrence features of the action.

To solve this problem, we propose a VAM based on ECA-Net. Through the VAM, we can find the important joints of the skeleton in each frame and establish the relationship of connection among them. These important vertices and their connection relationship constitute the global co-occurrence features of the skeleton in each frame. Specifically, the implementation of VAM can be divided into six steps:

1. Exchange of vertex dimension and channel dimension. We interchange the vertex dimension of the input data with its channel dimension by transpose function to realize the VAM by using ECN-Net. For example, let the shape of the tensor of input feature $f_{in}$ be $C_{in}\times T\times V$. Here, $C_{in}$ denotes the number of input channels and *T* denotes the number of frames in a video, while *V* denotes the total number of vertices in each frame. We transpose $f_{in}$ into $V\times T\times C_{in}$ to obtain a temp.
2. After being processed by ECA-NET, the shape of the temp is V×1.
3. The construction of an V×V matrix. The temp is multiplied by transposition of itself to obtain an V×V matrix $A^{v}$, which denotes the weight of each vertex and their interrelations. $A_{ij}^{v}$ represents the weight of the connection between $v_{i}$ and $v_{j}$, and if i=j, then $A_{ij}^{v}$ represents the weight of $v_{i}$.
4. The values of the matrix elements are normalized to 0–1 through the softmax layer.
5. The fusion of the V×V matrix and adjacency matrix. According to the vertex partition strategy of ST-GCN, the adjacency matrix of the graph is divided into three parts: $A_{i}$ (*i* = 1, 2, and 3). The sum of normalized $A^{v}$ and $A_{i}$ is assigned to $A_{i}^{{}^{\prime }}$.
6. Residual connection. The result of the matrix multiplication of the $A_{i}^{{}^{\prime }}$ and $f_{in}$ is embedded into $C_{out}\times T\times V$ via a 1×1 convolution, where $C_{out}$ denotes the number of output channels. If the number of input channels differs from the number of output channels, a 1×1 convolution is inserted into the residual path to transform the input so that it matches the output in the channel dimension. According to Equation (4), the calculation process of VAM can be written as follows:
  (5) $$\begin{array}{c} f_{in}^{l+1}=cov_{1\times 1}\left(\sigma \left(\sum_{i=1}^{3}\Lambda_{i}^{-\frac{1}{2}}A_{i}^{{}^{\prime }}\Lambda_{i}^{-\frac{1}{2}}f_{in}^{l}W^{l}\right)\right)+cov_{1\times 1}\left(f_{in}^{l}\right) \end{array}$$
  where $cov_{1\times 1}$ denotes a function of 1×1 convolution. The above implementation is shown in Figure 3.

### 4.3. Cross-kernel Feature Fusion (CFF)

Compared with CNN, the feature fusion process of previous related works [4,6,7] may not effectively extract the spatial features among adjacent nodes. It can be concluded that the feature fusion process of previous related works can be summarized in two steps: The first step is to expand the number of feature channels through 1×1 convolution. In the second step, GCN realizes the feature fusion among each vertex and its adjacent vertices. The plot (a) and plot (b) of Figure 4 show the feature fusion process of CNN and previous related work, respectively. In (a), the features represented by the blue cube in feature map can be written as: $f_{CNN}=\sigma \left(x1\times w1+x2\times w2+x4\times w3+x5\times w4\right)$, where $f_{CNN}$ not only represents the weights of the four features covered by the filter area but also represents the relative spatial position relationship of these four weights. In (b), the neighbor set composed of x2,x3, and x4, let x3 be a root node. Then, x3 in feature map_2 can be written as $f_{ST-GCN}=\sigma \left(x2\times w1+x3\times w1+x4\times w1\right)$. Here, $f_{ST-GCN}$ only involves one weight (w1), so it cannot contain the relative spatial position relationship of nodes in the neighbor set.

Besides, in hundreds of feature channels, there may be channels that are unrelated to motion recognition, which will affect the performance and robustness of the model. Different feature channels represent different features of action, such as posture features, motion features and offset features. For the action “walking”, posture features influence model performance due to poor quality frames. For the action "reading", offset features affect model performance due to camera shake. Therefore, we need to suppress the feature channels that affect the current action recognition to improve the robustness of the model.

To solve the above problems, we propose Cross-kernel Feature Fusion (CFF), consisting of Channel Attention Module (CAM) and Cross-kernel feature Fusion Algorithm (CFA). It can be seen from Figure 1b that in ST-GCN, the new feature of the root node in a neighbor set is only related to a filter in the previous layer, that is, the features of all nodes in a neighbor set are associated with the same convolution kernel. Our idea is to make these features related to different convolution kernels in the feature fusion process, that is, CFA. Through CFA, we can break through the limitations of previous related work. In order to suppress the influence of some feature channels on the model performance, we further introduce CAM. Figure 1c shows the overall structure of CFF by taking the graph composed of four nodes as an example, where the number of input feature channels and the number of output feature channels are 1. The overall workflow of CFF can be described as follows: Firstly, the number of the feature channels of the graph is expanded from 1 to 3 by three 1×1 filters, that is, feature map_1; Then the CAM recalibrates the size of each feature value to generate feature map_2, where the volume of each cube represents the weight of the feature channel in which it is located; Finally, feature fusion is realized by CFA. If the settings of the neighbor set and the root node are the same as (b), then the features of x3 in feature map_3 can be written as $f_{CFF}=\sigma \left(x3\times w1+x2\times w2+x4\times w3\right)$. Therefore, $f_{CFF}$ breaks through the limitation of $f_{ST-GCN}$, that is, it not only contains three weights but also contains the spatial position relation of these three weights. CAM and CFA will be described in detail below.

#### 4.3.1. Channel Attention Module (CAM)

By introducing the CAM, we can focus on the channels that are strongly related to the recognition task and restrain the channels that are not related to the action recognition task. The specific implementation process can be described as follows: Firstly, skeleton data can be described as a tensor with the shape C×T×V, where T denotes the number of frames of a video, V denotes the total number of nodes per frame, and C denotes the number of feature channels of each node. In the squeeze phase, the shape of the tensor is transformed into C×1×1 by global average pooling. In appropriate cross-channel interaction, the weight of each feature channel in dimension *C* is predicted by 1D convolution. Finally, with these weights, the tensor is recalibrated. Figure 5 shows this process.

#### 4.3.2. Cross-Kernel feature Fusion Algorithm (CFA)

Our motivation is to make each node in a neighbor set have different weights, to break through the limitation of ST-GCN feature fusion, and then effectively extract the spatial features among adjacent nodes. As shown in Figure 4c, through CFF, the feature of x3 in feature map_3 can be written as follows: $f_{3}=\sigma \left(x3\times w1+x2\times w2+x4\times w3\right)$. So, x2,x3, and x4 in the neighbor set correspond to w1, w2, and w3 respectively. However, we do not answer how this process is realized. Therefore in this section, we focus on the implementation algorithm of CFF, namely CFA. The implementation process can be divided into six steps:

1. Given a graph, the number of feature channels of the graph, and the number of output feature channels, we find the largest neighbor set through the function: $g_{num}=max\left\{N_{1},N_{2},\cdots ,N_{n}\right\}$, where $g_{num}$ denotes the cardinality of the largest neighbor set. $N_{i}$ denotes the number of adjacent vertices of the $i^{\mathrm{th}}$ vertex (including vertex i itself), *n* refers to the number of the vertex in a graph.
2. We determine the number of 1×1 filters according to the function: $fil_{num}=g_{num}\times C_{out}$, where $C_{out}$ denotes the number of output feature channels.
3. Through $fil_{num}$ filters, the number of feature channels of the graph is extended to $fil_{num}$.
4. All node features are divided into $g_{num}$ groups, each of which contains $C_{out}$ feature channels of each node.
5. The division of the adjacency matrix of the graph. This division process can be divided into three steps: The first step, the diagonal matrix representing the vertex itself is separated from the adjacency matrix; The second step, a matrix composed of one non-zero element in each column of the adjacency matrix is separated from the adjacency matrix, and the position of the non-zero elements of the matrix is the same as its position in the adjacency matrix; The third step, repeat the second step until the non-zero elements in each column of the adjacency matrix are separated. By this partition algorithm, the adjacency matrix of the graph is divided into $g_{num}$ matrices.
6. Let these $g_{num}$ groups of feature be expressed as $x=\{x1,x2,\cdots ,x_{g_{num}}\}$, and these $g_{num}$ matrices can be expressed as $A=\{A_{1},A_{2},\cdots ,A_{g_{num}}\}$. Then *x* and *A* perform matrix multiplication, which can be described as $f_{out}=\sum_{j}^{g_{num}}x_{j}A_{j}$.

To describe the above CFA more clearly, we describe the implementation process of CFA in Figure 4c and Figure 6 with an example of a graph containing four nodes. According to the graph in the input of Figure 4c, we can get $g_{num}=3$. Let $C_{out}=1$, then the number of filters $fil_{num}=3\times 1=3$. So, in the input of Figure 4c, we use three small cubes of different colors to represent the three filters. Through these three filters, the number of feature channels of the graph in the input of Figure 4c is expanded from 1 to 3, i.e., Figure 6a. Because $g_{num}$ = 3, the features of all nodes and the adjacency matrix of this graph are divided into three, which are shown in Figure 6b. Please note: Since $C_{out}=1$, each group of features is composed of one feature channel for each node. Let three groups of features and three matrices be: $x_{1},x_{2},x_{3},A_{1},A_{2}$, and $A_{3}$ respectively, then the feature fusion process can be expressed as $f_{out}=\sum_{j}^{3}x_{j}A_{j}$, which is shown in Figure 6c. In Figure 6e, these white numbered cubes represent the root node, where the feature of the root node 3 can be written as follows: $f_{3}=\sigma \left(x3\times w1+x2\times w2+x4\times w3\right)$. Since w1,w2, and w3 come from different convolution kernels, CFA is realized.

### 4.4. GCLS

As introduced in Section 1, VAM-branch and CFF-branch are fused by the voting mechanism. In detail, we first obtain bone data according to the method in 2s-AGCN [7]. Then, the joint data and bone data are fed into the VAM-branch and CFF-branch, respectively. Finally, the softmax scores of the two branches are added to obtain the fused score by which we predict the action label.

## 5. Experiments

We evaluate the effectiveness of Global Co-occurrence feature and Local Spatial feature learning model (GCLS) on two benchmark datasets. In-depth analyses are made on the NTU-RGB+D. To better understand the model, visualization for the joints and bones of skeleton are given.

### 5.1. Datasets and Model Configuration

In this section, according to the characteristics set of the comparison datasets proposed by R.Singh et al. [36], NTU-RGB+D and Kinetics-Skeleton are selected. At the same time, we give a brief introduction of the two data sets respectively. Finally, we show the hyperparameters of the model on the two datasets.

#### 5.1.1. NTU-RGB+D

**Figure 7:**
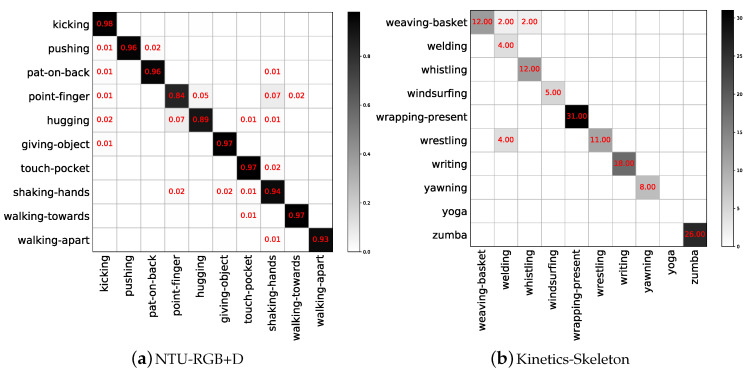
Local graphs of confusion matrices of the NTU-RGB+D and Kinetics-Skeleton for action recognition.

The NTU-RGB+D [17] dataset is so far the largest skeleton-based human action recognition dataset. It contains 56880 skeleton sequences. Each of these sequences is annotated as one of 60 action classes. There are two recommended evaluation protocols, namely Cross-Subject (CS) and Cross-View (CV). In the Cross-Subject setting, sequences of 20 subjects are used for training, while sequences of the remaining 20 subjects are used for validation. In the Cross-View setting, samples are split by camera views. Samples from two camera views are used for training and the rest are used for testing. The confusion matrix of the validation set for NTU-RGB+D is shown in Figure 7a where the accuracy is 96.1%. The experiment represented by this matrix is based on Cross-View. Due to a large number of action categories, Figure 7a only shows the part of the confusion matrix.

#### 5.1.2. Kinetics-Skeleton

Kinetics-400 [37] consists of ∼240 k training videos and 20 k validation videos in 400 human action categories. Because the dataset does not provide the skeleton information of the video, we obtain 2D skeleton data by estimating joint locations on certain pixels with the OpenPose toolbox [38]. In the multi-person video clip, we choose the two people for whom the confidence in the skeleton joint coordinate information is the highest as the input data of our model. To make the experimental results more comparable with other advanced algorithms, we use the same training and testing methods as related research work; that is, training our model on the training set, and reporting the accuracy of top-1 and top-5 on the verification set. Due to the poor quality of the skeleton in Kinetics-Skeleton, our accuracy was only 37.5%. The confusion matrix in Figure 7b reflects this phenomenon where Yoga poses are completely unrecognizable. Since there are as many as 400 action categories in this dataset, Figure 7b only shows the part of the confusion matrix, and the values in this matrix are not normalized.

#### 5.1.3. Model Setting

All experiments are conducted on PyTorch 1.2.0 and GeForce RTX 2080Ti GPUs. For NTU-RGB+D, we use the SGD algorithm to train the two branches of GCLS for 60 epochs. The learning rate of VCM-branch is initially 0.075, decaying by 0.1 every 10 epochs. Moreover, the learning rate of CFF-branch is initially 0.085, which is divided by 10 at the 30th epoch and 40th epoch. For Kinetics, we also use the SGD algorithm to train the two branches of GCLS for 65 epochs. The initial learning rates of both branches are 0.085, which are divided by 10 at the 45th epoch and 55th epoch.

**Figure 8:**
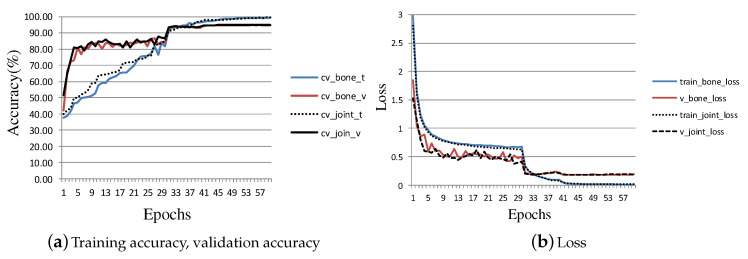
The training accuracy, validation accuracy and loss achieved at each epoch during the training process of the CFF-branch for NTU-RGB+D.

Specifically, Figure 8 shows the changes of training accuracy, verification accuracy and loss in the process of training CFF-branch. Figure 8a shows the variation of training accuracy and verification accuracy, where cv_bone_t and cv_bone_v represent the training accuracy and verification accuracy with bone as input data, respectively. The prefix cv indicates that these experiments are based on Cross-View. The other two lines represent the training accuracy and verification accuracy when the input data are joints. Figure 8b shows how the losses change when the experiments of Figure 8a are carried out. Here, train_bone_loss and v_bone_loss represent the training loss and verification loss when the input data are bones. The other two lines represent the losses when the input data are joints. It can be seen from Figure 8a,b that the losses and accuracy have changed significantly at the 31st epoch and 41st epoch, which is caused by the reduction of the learning rate at the 30th epoch and 40th epoch.

### 5.2. Comparison with the State-of-the-Art

We compared the performance of our model with state-of-the-art models based on skeleton motion recognition on the datasets of NTU-RGB+D and Kinetics. These comparisons are presented in Table 1 and Table 2, respectively. In Table 1, we divide the comparison models into four categories: traditional method [9], RNN-based methods [12,14,15,16,17,39,40], CNN-based methods [8,27], and GCN-based methods [4,6,7,41,42,43]. Although 2s Shift-GCN [43] performs slightly better on Cross-subject benchmark of the NTU-RGB+D than our model, it is tailored for the 3D skeleton and can not be effectively applied to the 2D skeleton. The accuracy of DGNN [41] on Cross-subject benchmark of the NTU-RGB+D is slightly higher than our model by 0.4%, but the DGNN uses four input data streams while our model only uses two input data streams, and its calculation cost is 16.4 times more than our model.

In the Kinetics dataset, we compare our model with eight state-of-the-art approaches. These eight approaches can be divided into four categories: traditional method [44], LSTM-based method [17], CNN-based method [24], and GCN-based methods [4,6,7,41,42]. Table 2 presents the top-1 and top-5 classification performances.

Above, we compared our model with related advanced models from the perspective of accuracy. Next, we compare our model with related models in terms of efficiency, spatial complexity, and time complexity, which are shown in Table 3. Here, Params, Flops, and Inference time measure spatial complexity, time complexity, and efficiency respectively, where ST-GCN and 2s-AGCN were regarded as the baseline methods and the recent competition respectively.

### 5.3. Ablation Study

To analyze the performance of each component of our model, we conducted extensive experiments on the Cross-View benchmark of the NTU-RGB+D [17].

**Figure 9:**
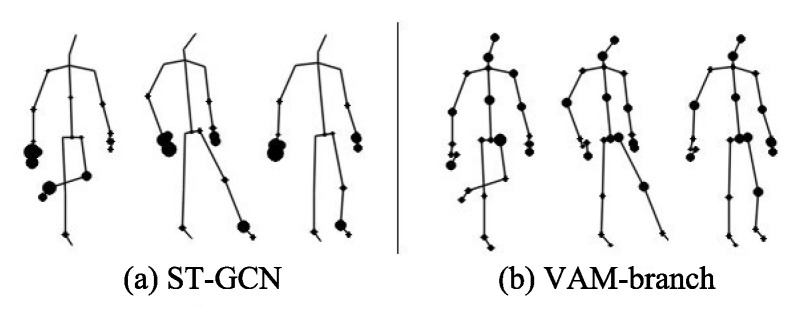
The global co-occurrence features comparison of joints between ST-GCN and VAM-branch.

**Effect of VAM-branch:** While ST-GCN tries to aggregate wider-range features in hierarchical GCNs, node features might be weakened during the long diffusion [5]. Therefore, ST-GCN is unable to effectively obtain the global co-occurrence features of all vertices of the skeleton. Vertex attention mechanisms can effectively solve this problem. The left columns of Table 4 present the results of the performance comparison between the original ST-GCN model and VAM-branch, which shows that the performance is improved by 3.3%. In Figure 9, we visualized features of vertices of “kicking something” action in the NTU-RGB+D, which further intuitively described the comparison results in Table 4. As can be observed in Figure 9, VAM-branch can effectively extract global co-occurrence features.

**Figure 10:**
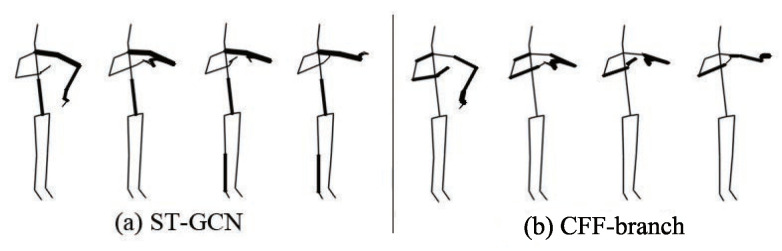
The spatial structure features comparison of bones between ST-GCN and CFF-branch.

**Effect of CFF-branch:** CFF-branch is mainly composed of Channel Attention Module (CAM) and Cross-kernel feature Fusion Algorithm (CFA). We analyze the performance of each module. The model performance after removing CAM and CFA respectively is detailed in the middle columns of Table 4. Without CAM, performance decreases by 1.3%; Without CFA, moreover, performance reduces by 1.7%. Figure 10 shows the feature maps of action ‘tear up paper’ in the ST-GCN model and CFF-branch. ST-GCN only captures the spatial structural features of a single-arm, while CFF-branch effectively captures the spatial structure features of double arms. CAM makes the CFF-branch pay more attention to the features most relevant to action recognition and ignores other features; thus, it overlooks the features of the trunk and legs in Figure 10.

**Effect of voting mechanism:** Another important reason for the performance improvement of the model is that we design a network composed of the VAM-branch and the CFF-branch. The right columns of Table 4 show the accuracy of VAM-branches and CFF-branches, with joints and bones as input data respectively, along with the accuracy after integration of the two branches.

## 6. Conclusions

We propose a Global Co-occurrence feature and Local Spatial feature learning model (GCLS), which consists of two branches, for skeleton-based action recognition. The Vertex Attention Mechanism branch (VAM-branch) focuses on the extraction of global co-occurrence features, while the Cross-kernel Feature Fusion branch (CFF-branch) focuses on the extraction of spatial structure features composed of adjacent bones and the filtering of channels unrelated to action recognition. Through the combination of co-occurrence feature and adjacency matrix, we can obtain the connection of any two important nodes, which realizes the information transmission between the two nodes. In other words, VAM-branch improves the accuracy of action recognition by improving the model’s ability to capture the dependency between far-apart joints. The CFF is composed of a Channel Attention Module (CAM) and a Cross-kernel feature Fusion Algorithm (CFA). The CAM improves the robustness of the model by filtering the channel independent of the current action. By making the features of each node in the neighbor set relate to different convolution kernels, the CFA has realized the effective acquisition of local spatial structure features composed of adjacent bones. The co-occurrence feature focuses on the global representation of the action, while the local spatial feature focuses on the local detail representation of the action. We integrate the two features through the voting mechanism to further improve the performance of the model. Experiments on the NTU-RGB+D and the Kinetics demonstrate that our method can fully capture the global co-occurrence features and spatial structure features, and can also achieve better performance than state-of-the-art works. When there are more than three people in the scene, the performance of the model will be greatly reduced, which is the problem we need to solve in the future.

## Figures and Tables

**Table 1:** Comparison of action recognition performance with state-of-the-art methods on the Kinetics dataset.

Methods	TOP-1 (%)	TOP-5 (%)
Feature Enc [44] (2015)	14.9	25.8
Deep LSTM [17] (2016)	16.4	35.3
TCN [24] (2017)	20.3	40.0
ST-GCN [4] (2018)	30.7	52.8
AS-GCN [6] (2019)	34.8	53.5
2s-AGCN [7] (2019)	36.1	58.7
DGNN [41] (2019)	36.9	59.6
Search-based GCN [42] (2020)	37.1	60.1
GCLS (our)	**37.5**	**60.5**

**Table 2:** Comparison of action recognition performance with state-of-the-art methods on the NTU-RGB+D.

Methods	CS (%)	CV (%)
Lie Group [9] (2014)	50.1	52.8
H-RNN [15] (2015)	59.1	64.0
Deep LSTM [17] (2016)	60.7	67.3
ST-LSTM+TS [14] (2016)	69.2	77.7
STA-LSTM [12] (2017)	73.4	81.2
VA-LSTM [39] (2017)	79.2	87.7
ARRN-LSTM [16] (2018)	80.7	88.8
Ind-RNN [40] (2018)	81.8	88.0
CNN+Motion+Trans [27] (2017)	83.2	89.3
DPRL [45] (2018)	83.5	89.8
HCN [8] (2018)	86.5	91.1
ST-GCN [4] (2018)	82.6	89.6
AS-GCN [6] (2019)	86.8	94.2
2s-AGCN [7] (2019)	88.5	95.1
DGNN [41] (2019)	89.9	96.1
Search-based GCN [42] (2020)	89.4	95.7
2s Shift-GCN [43] (2020)	89.7	96.0
GCLS (our)	**89.5**	**96.1**

**Table 3:** Comparison of spatial complexity, time complexity and efficiency.

Model	Params (M)	Flops (G)	Taining Time (h)	Inference Time (ms)	Conference
ST-GCN	2.47	6.21	46.67	5.20	AAAI2018
2s-AGCN	4.01	7.99	47.38	6.47	CVPR2019
GCLS (our)	3.08	7.73	44.30	6.10	

**Table 4:** Performance comparison of each component of our model. wo/X means deleting the X module.

Performance of VAM		Performance of CFA and CAM		Merging of Two Branches	
**Model**	**Accuracy (%)**	**Components of CFF**	**Accuracy (%)**	**Modular**	**Accuracy (%)**
ST-GCN	91.7	CFF-branch wo/CFA	93.1	VAM-branch	95.0
VAM-banches	95.0	CFF-branch wo/CAM	93.5	CFF-branch	94.8
		CFF-branch	94.8	GCLS	96.1
